# Genomic Analysis Reveals the Genetic Determinants Associated With Antibiotic Resistance in the Zoonotic Pathogen *Campylobacter* spp. Distributed Globally

**DOI:** 10.3389/fmicb.2020.513070

**Published:** 2020-09-11

**Authors:** Daniel Rivera-Mendoza, Irma Martínez-Flores, Rosa I. Santamaría, Luis Lozano, Víctor H. Bustamante, Deyanira Pérez-Morales

**Affiliations:** ^1^Programa de Maestría en Biotecnología, Centro de Investigación en Biotecnología, Universidad Autónoma del Estado de Morelos, Cuernavaca, Mexico; ^2^Programa de Genómica Evolutiva, Centro de Ciencias Genómicas, Universidad Nacional Autónoma de México, Cuernavaca, Mexico; ^3^Departamento de Microbiología Molecular, Instituto de Biotecnología, Universidad Nacional Autónoma de México, Cuernavaca, Mexico; ^4^CONACYT-Centro de Investigación en Biotecnología, Universidad Autónoma del Estado de Morelos, Cuernavaca, Mexico

**Keywords:** *Campylobacter*, zoonotic bacteria, antibiotic resistance, resistance genes, genomic analysis, resistome

## Abstract

The genus *Campylobacter* groups 32 Gram-negative bacteria species, several being zoonotic pathogens and a major cause of human gastroenteritis worldwide. Antibiotic resistant *Campylobacter* is considered by the World Health Organization as a high priority pathogen for research and development of new antibiotics. Genetic elements related to antibiotic resistance in the classical *C. coli* and *C. jejuni* species, which infect humans and livestock, have been analyzed in numerous studies, mainly focused on local geographical areas. However, the presence of these resistance determinants in other *Campylobacter* species, as well as in *C. jejuni* and *C. coli* strains distributed globally, remains poorly studied. In this work, we analyzed the occurrence and distribution of antibiotic resistance factors in 237 *Campylobacter* closed genomes available in NCBI, obtained from isolates collected worldwide, in different dates, from distinct hosts and comprising 22 *Campylobacter* species. Our data revealed 18 distinct genetic determinants, genes or point mutations in housekeeping genes, associated with resistance to antibiotics from aminoglycosides, β-lactams, fluoroquinolones, lincosamides, macrolides, phenicols or tetracyclines classes, which are differentially distributed among the *Campylobacter* species tested, on chromosomes or plasmids. Three resistance determinants, the *bla*_OXA–493_ and *bla*_OXA–576_ genes, putatively related to β-lactams resistance, as well as the *lnu*(AN2) gene, putatively related to lincosamides resistance, had not been reported in *Campylobacter*; thus, they represent novel determinants for antibiotic resistance in *Campylobacter* spp., which expands the insight on the *Campylobacter* resistome. Interestingly, we found that some of the genetic determinants associated with antibiotic resistance are *Campylobacter* species-specific; e.g., the *bla*_OXA–493_ gene and the T86V mutation in *gyrA* were found only in the *C. lari* group, whereas genes associated with aminoglycosides resistance were found only in *C. jejuni* and *C. coli*. Additional analyses revealed how are distributed the resistance and multidrug resistance *Campylobacter* genotypes assessed, with respect to hosts, geographical locations, and collection dates. Thus, our findings further expand the knowledge on the factors that can determine or favor the antibiotic resistance in *Campylobacter* species distributed globally, which can be useful to choose a suitable antibiotic treatment to control the zoonotic infections by these bacteria.

## Introduction

The discovery and consequent therapeutic use of antibiotics was a remarkable advance in human medicine, which prevented the mortal outcomes of bacterial infections, saving millions of lives during the last century. However, bacteria have evolved through diverse mechanisms, intrinsic and acquired, to withstand the harmful activity of antibiotics. The antibiotic resistance (AR) is mainly generated by the presence of specific resistance genes or point mutations in some housekeeping genes; likewise, the AR can be transferred between bacteria through different mechanisms of DNA exchange, which has greatly increased the occurrence and spread of antibiotic-resistant bacteria worldwide. The development of AR has progressively compromised the effective use of antibiotics, restricting the therapeutic options available to treat the illness produced by antibiotic-resistant pathogenic bacteria. Nowadays, pathogenic bacteria that show resistance to a great diversity of antibiotics represent a serious threat to health worldwide. It has been estimated that infections produced by these bacteria could cause 10 million annual deaths by 2050 (O’Neill, 2014). Faced with this risk to human health, the World Health Organization (WHO) issued a priority global list of antibiotic-resistant pathogenic bacteria for which there is an urgent need to direct research for discovery and development new antibiotics (WHO, 2017). Importantly, in this WHO report, and also in a published analysis from the United States Centers for Disease Control and Prevention (CDC), antibiotic-resistant *Campylobacter* spp. were cataloged as a serious health hazard in the world (CDC, 2013; WHO, 2017).

The genus *Campylobacter* groups biologically diverse species. These are Gram-negative, chemoorganotrophic, non-sporeforming epsilonproteobacteria. Depending on the species, these can be slender, spirally curved-, curved- or straight- rods; with a single polar flagellum, bipolar or multiple flagella, or no flagellum; and microaerobic or anaerobic bacteria (Vandamme et al., 2015). At the time of writing this paper, the genus *Campylobacter* comprised 32 species and 13 subspecies with validly published names^1^. Twenty *Campylobacter* species have been isolated from symptomatic or healthy humans: *C. coli*, *C. concisus*, *C. curvus*, *C. fetus*, *C. gracilis*, *C. helveticus*, C. *hominis*, *C. hyointestinalis*, *C. insulaenigrae*, *C. jejuni*, *C. lanienae*, *C. lari*, *C. mucosalis*, *C. peloridis*, *C. rectus*, *C. showae*, *C. sputorum*, *C. upsaliensis*, *C. ureolyticus* and *C. volucris* (Man, 2011; Kweon et al., 2015). Some of these *Campylobacter* species have also been isolated from the gastrointestinal tract of animals, mainly farm animals (poultry, pigs, cattle, and sheep), where *Campylobacter* spp. reside usually as commensal microorganisms (Silva et al., 2011).

In humans, *Campylobacter* spp. can cause campylobacteriosis, which is considered the leading food-borne zoonosis and the most common cause of gastroenteritis in the world (EFSA and ECDC, 2018)^2^. *C. jejuni* and *C. coli* are the *Campylobacter* species more frequently involved in human gastroenteritis, hence, these two species have been by far the most studied (Man, 2011; Kaakoush et al., 2015). However, other species such as *C. concisus*, *C. lari*, *C. upsaliensis*, and *C. ureolyticus*, have also begun to be recognized as causative agents of human and animal campylobacteriosis; therefore, they are known as emerging *Campylobacter* species (Man, 2011).

In 2010, the global burden of *Campylobacter* infections was 95,613,970 clinical cases; 21,374 deaths and 2,141,926 DALYs (Disability Adjusted Life Years) (Havelaar et al., 2015). Campylobacteriosis may cause mild to severe clinical signs, or even be asymptomatic. The common symptoms of *Campylobacter* enteric infections include diarrhea (often bloody), fever, abdominal cramps, headache, nausea and/or vomiting. In vulnerable populations, such as very young children, elderly or immunologically compromised patients, this illness can be mortal^2^. Furthermore, other gastrointestinal manifestations or severe life threatening extragastrointestinal complications may appear (Man, 2011; Kaakoush et al., 2015). Because most *Campylobacter* enteric infections are self-limiting, antibiotic administration is usually not required. Antibiotic therapy is recommended in patients with severe clinical symptoms, relapses, or a prolonged course of infection (Tang et al., 2017). In these cases, fluoroquinolones such as ciprofloxacin, and macrolides such as erythromycin, are the drugs of choice (Ge et al., 2013).

A rapid and constant increase in the frequency of antibiotic-resistant *Campylobacter* strains isolated from humans and animals has been recognized worldwide (Luangtongkum et al., 2009; Cody et al., 2010; Tang et al., 2017; Signorini et al., 2018). It has been reported a wide-ranging prevalence of *Campylobacter* strains resistant to the following antibiotic families: aminoglycosides, β-lactams, cephalosporins, fluoroquinolones, fosfomycins, lincosamides, macrolides, phenicols, quinolones, sulfonamides, and tetracyclines (Ishihara et al., 2004; Karikari et al., 2017; Lee et al., 2017; Premarathne et al., 2017; Agunos et al., 2018; Bailey et al., 2018; Ewers et al., 2018; Iglesias-Torrens et al., 2018; Khan et al., 2018; Signorini et al., 2018; Wei and Kang, 2018; Zhang et al., 2018; Nowaczek et al., 2019; Schiaffino et al., 2019). Moreover, a prevalence of up to 94% of multidrug resistant (MDR; resistant to three or more antibiotic families) *Campylobacter* isolates, in different parts of the world, has been reported (Zhang et al., 2018). A lot of relevant information about the genetic determinants mediating AR in *C. coli* and *C. jejuni* has been reported (Taylor and Courvalin, 1988; Payot et al., 2006; Alfredson and Korolik, 2007; Luangtongkum et al., 2009; Smith and Fratamico, 2010; Iovine, 2013; Tang et al., 2017; Shen et al., 2018); however, the genetic determinants for AR in the rest of the *Campylobacter* species, including the emerging species, are greatly unknown.

By its importance for public health and food safety, it is necessary to know the resistome of the genus *Campylobacter*; the resistome is defined as the collection of AR determinants in a specific bacteria or ecological niche (D’Costa et al., 2006; Wright, 2007; Hu et al., 2017). A very high correlation between the genotype and phenotype for AR has been observed in *Campylobacter* (Zhao et al., 2016; de Vries et al., 2018; Whitehouse et al., 2018). Therefore, the identification of the *Campylobacter* genotypes associated with AR could help to choose the best antibiotic treatment against infections by *Campylobacter* species.

The aim of this study was to gain insight into the genetic determinants that constitute the *Campylobacter* resistome.

## Materials and Methods

### Bacterial Genomes

A total of 237 closed genomes (chromosome and plasmid) of *Campylobacter* spp. were retrieved from the open-access RefSeq: NCBI Reference Sequence Database^3^ in March 2019. NCBI accession numbers of the 237 genomes, the information about the host, collection date and geographic location of the strains from which the DNA was extracted, sequenced and annotated, as well as the number of plasmids annotated as an assembly unit, are registered in Supplementary File S1.

### *In silico* Identification of Genes Associated With Antibiotic Resistance

Datasets from publicly available resistance gene databases Comprehensive Antibiotic Resistance Database (CARD^4^) (Jia et al., 2017) and the National Database of Antibiotic Resistant Organisms (NDARO^5^), were downloaded (March 2019) and used to identify the presence of genes associated with AR in the 237 *Campylobacter* genomes, by following two different approaches. In the first one, the BPGA software (Chaudhari et al., 2016) was applied to clustering the resistance genes from CARD and NDARO with those of the *Campylobacter* genomes, using the USEARCH clustering tool with default parameters (a cutoff set of 50% amino acid identity and 20 random permutations). In the second approach, a BLASTp search was performed^6^ with all predicted ORFs from the *Campylobacter* genomes, against the products of resistance genes from CARD and NDARO, using an *E* value cutoff of 10E-5, a selected threshold of 50% amino acid identity and minimum coverage of 60% of the query sequence length. Both approaches were compared to ensure the identification of the respective gene associated with AR, on the base of the best hit.

### *In silico* Identification of Point Mutations Associated With Antibiotic Resistance

Specific point mutations known to mediate resistance to fluoroquinolones (*gyrA*), macrolides (*rplD*, *rplV* and 23S rRNA) and streptomycin (*rpsL*), in *C. coli* or *C. jejuni*, were sought in the 237 *Campylobacter* spp. genomes by using publicly available ResFinder database version 3.2^7^ (April 2019) (Zankari et al., 2012). To confirm the point mutations identified by ResFinder, the amino acid sequence of GyrA or RpsL, as well as the nucleotide sequence of 23S rRNA, were aligned and examined using the MEGA X software version 10.1 with the MUSCLE algorithm^8^.

## Results

### Identification of Genetic Determinants Associated With AR in *Campylobacter*

Genetic elements associated with AR, i.e., specific genes and point mutations in housekeeping genes, were sought by *in silico* analysis in a total of 237 closed publicly available *Campylobacter* genomes, as described in the “Materials and Methods” section. The 237 genomes assessed spanned 22 species of *Campylobacter*: *C. avium* (1), *C. coli* (22), *C. concisus* (3*), C. cuniculorum* (1), *C. curvus* (1), *C. fetus* (11), *C. gracilis* (1), *C. helveticus* (1), *C. hepaticus* (1), *C. hominis* (1), *C. hyointestinalis* (2), *C. iguaniorum* (3), *C. insulaenigrae* (3), *C. jejuni* (163), *C. lanienae* (1), *C. lari* (8), *C. peloridis* (1), *C. pinnipediorum* (5), *C. sputorum* (4), *C. subantarcticus* (2), *C. ureolyticus* (1) and *C. volucris* (1); the number of genomes tested for each species is indicated between parenthesis. Most genomes analyzed were from *C. jejuni* (68.8%) and *C. coli* (9.3%); genomes from the 20 remaining *Campylobacter* species denoted 21.9% of the total. Important to note, in our analysis, we considered a genome as that including the sequence of both the chromosome and plasmids, when present, from the respective strain.

**FIGURE 1:**
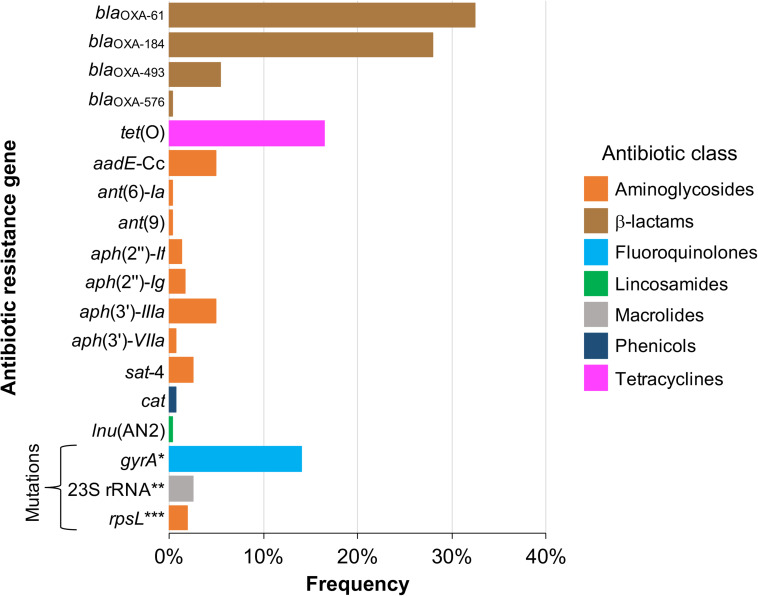
Frequency of genetic determinants associated with antibiotic resistance on *Campylobacter* genomes. Genetic determinants for antibiotic resistance (resistance genes and mutations in housekeeping genes targets of antibiotics) present in 237 closed publicly available genomes belonging to 22 *Campylobacter* species, were identified by *in silico* analysis as described in “Materials and Methods” section. The colors indicate the antibiotic class to which the respective genetic determinant putatively confers resistance. *T86I, T86K, T86V, P104T or T86A/D90Y substitutions; **A2074G or A2074G/A2075G point mutations; ***K43R, K88R or K43R/K88R substitutions.

As depicted in Figure 1, 15 acquired genes associated with resistance to 5 distinct antibiotic classes were identified. Those more frequently found were *bla*_OXA–61_ and *bla*_OXA–184_, showing a prevalence of 32.5% (77/237 genomes) and 27.8% (66/237 genomes), respectively, both coding class D oxacillinase (OXA)-type β-lactamases (Figure 1). Acquired resistance to some β-lactams antibiotics has been associated with β-lactamases production in many organisms, including *Campylobacter* (Taylor and Courvalin, 1988; Alfredson and Korolik, 2005; Griggs et al., 2009). Notably, our analysis revealed 2 additional putative OXA-type β-lactamase genes, *bla*_OXA–493_ (prevalence of 5.49%; 13/237 genomes) and *bla*_OXA–576_ (prevalence of 0.42%; 1/237 genomes) (Figure 1), which are annotated in the respective genomes, but had not been previously reported. All these genes for β-lactamases are located on chromosome (Table 1), which is consistent with the fact that plasmids coding β-lactamases have not been described in *Campylobacter*.

**TABLE 1:** Location (chromosome or plasmid) of 15 antibiotic resistance genes detected on *Campylobacter* closed genomes.

Antibiotic class	Gen	Genomes^a^	Location	
			Chromosome	Plasmid
β-lactams	*bla*_*OXA–*61_	77	77	0
β-lactams	*bla*_*OXA–*184_	66	66	0
β-lactams	*bla*_*OXA–*493_	13	13	0
β-lactams	*bla*_*OXA–*576_	1	1	0
Tetracyclines	*tet*(O)	39	9	30
Phenicols	*cat*	2	2	0
Lincosamides	*lnu*(AN2)	1	0	1
Aminoglycosides	*aph*(2″)-*If*	3	2	1
Aminoglycosides	*aph*(2″)-*Ig*	4	0	4
Aminoglycosides	*ant*(6)-*Ia*	1	0	1
Aminoglycosides	*aadE-C*c	12	12	0
Aminoglycosides	*ant*(9)	1	0	1
Aminoglycosides	*aph*(3′)-*IIIa*	12	0	12
Aminoglycosides	*aph*(3′)-*VIIa*	2	0	2
Aminoglycosides	*sat*-4	6	0	6

*^*a*^Number of genomes where the genes were located.*

**FIGURE 2:**
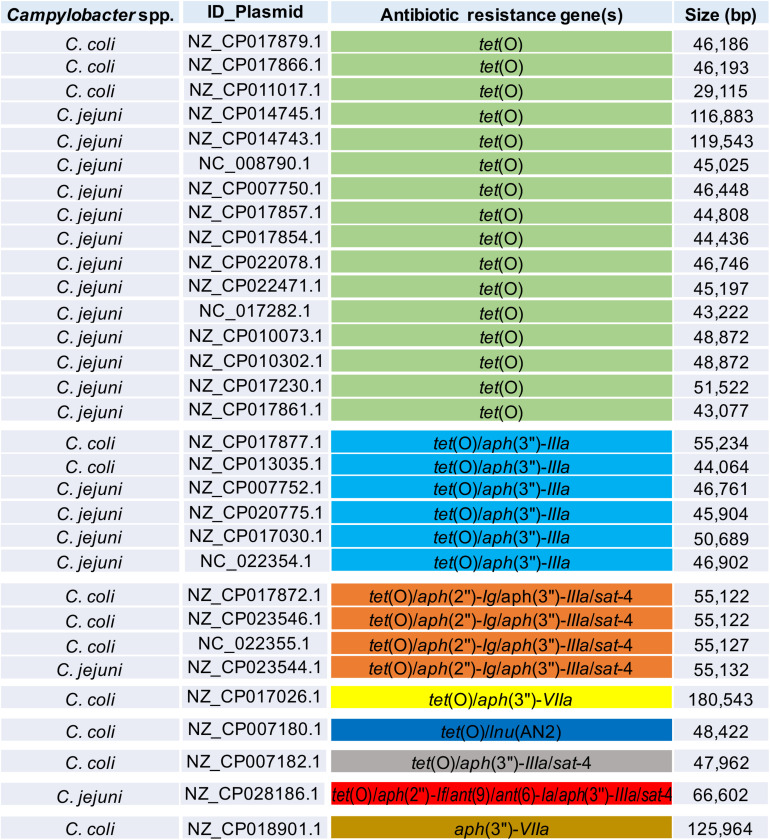
Antibiotic resistance patterns from *Campylobacter* plasmids. *Campylobacter* species harboring the respective plasmid, NCBI accession number for each plasmid, genes for antibiotic resistance, and size (bp) for each plasmid, are indicated. Distinct antibiotic resistance patterns are displayed in different color.

In addition to genes for β-lactamases, we found that 16.5% of analyzed genomes (39/237 genomes) harbor the *tet*(O) gene (Figure 1), which codes for the ribosomal protection protein involved in resistance to tetracyclines (Sougakoff et al., 1987). Consistent with previous reports indicating the presence of *tet*(O) either on plasmid or chromosome (Pratt and Korolik, 2005; Dasti et al., 2007), we found that 30 and 9 of the analyzed genomes carry this resistance gene on plasmid and chromosome, respectively (Table 1). It should be noted that *Campylobacter* plasmids are usually classified according to the genes they carry, those harboring the *tet*(O) gene have been called pTet plasmids (Marasini et al., 2018). From 87 plasmids annotated as an assembly unit separated from chromosome in the total of *Campylobacter* genomes tested, 30 were pTet and only one plasmid containing an AR gene other than *tet*(O) was found; the 31 plasmids harboring AR genes show sizes ranging from 29,115 to 180,543 bp (Figure 2). These pTet plasmids carry the *tet*(O) gene alone or together with other AR genes; interestingly, all of them, including that no pTet, are present only on genomes from *C. coli* and *C. jejuni* (Figure 2).

**FIGURE 3:**
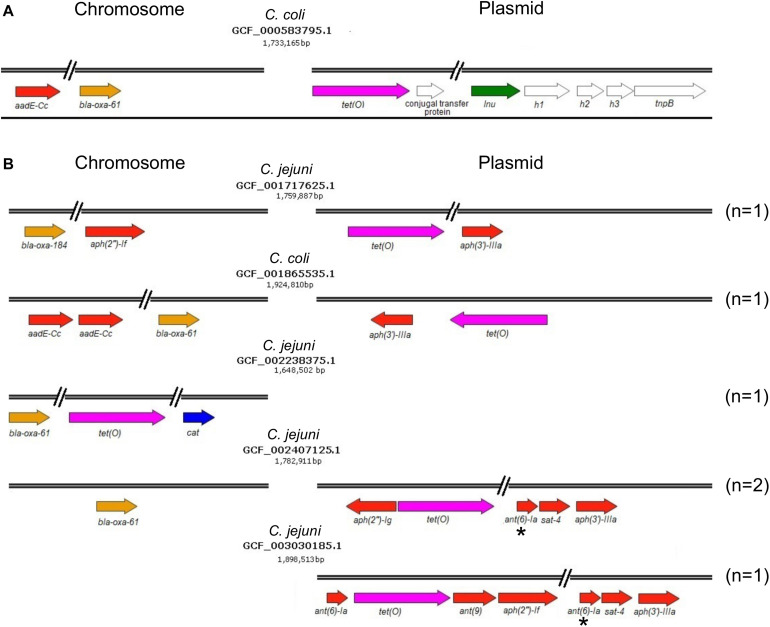
Resistance or multidrug resistance genotypes harbored on genomes of *Campylobacter* spp. **(A)** Multidrug resistance genotype containing the *lnu*(AN2) gene on a 48,422 bp plasmid from the *C. coli* RM5611 isolate. **(B)** Resistance or multidrug resistance genotypes containing different combinations of genetic determinants associated with antibiotic resistance on chromosome and/or plasmid. The name of the *Campylobacter* species from which the respective genome was obtained, as well as the NCBI accession number and size (bp) of genomes are shown. The colors indicate the antibiotic class to which the respective genetic determinant putatively confers resistance. Brown, β-lactams; pink, tetracyclines; blue, phenicols; green, lincosamides; and red, aminoglycosides. The genes shown with white color are not related to antibiotic resistance. The asterisks indicate truncated genes. The number (n) of genomes harboring each antibiotic resistance genotype is specified on the right. Arrows denote the direction of transcription for each gene.

On another hand, genes conferring resistance to phenicols or lincosamides were detected in a very small proportion of the analyzed genomes. Only 2 genomes harbor the *cat* gene (prevalence of 0.84%), which codes for a chloramphenicol acetyltransferase that is associated with resistance to chloramphenicol (Wang and Taylor, 1990); in both cases, this gene was located on chromosome (Figure 1 and Table 1). Regarding lincosamides resistance, a putative *lnu*(AN2) gene, coding an *O*-nucleotidyltransferase that mediates resistance to lincomycin (Wang et al., 2000), was only identified in one genome (prevalence of 0.42%) (Figure 1). Previous reports detected the *lnu*(C) and *lnu*(D) lincosamides resistance genes in genomes from *Campylobacter* (Zhao et al., 2016; Fabre et al., 2018), but to the best of our knowledge, this is the first study identifying the *lnu*(AN2) gene in this genus; specifically in the *C. coli* strain RM5611. The deduced amino acid sequence from this *lnu*(AN2) gene shares 64% identity to that from the *lnu*(AN2) gene of *Bacteroides fragilis* (NCBI Reference Sequence: NG_047920.1). In *B. fragilis*, this gene is found on chromosome; in contrast, in *C. coli* RM5611 the *lnu*(AN2) gene is located on a plasmid of 48,422 bp, which also contains the *tet*(O) gene (Figure 3A). Comparative analysis indicated that there is not synteny for the *lnu*(AN2) gene between *C. coli* and *B. fragilis* (data not shown).

Furthermore, our analysis also identified 8 different genes that code for distinct aminoglycoside-modifying enzymes associated with resistance to diverse aminoglycosides: *aph*(2″)-*If* and *aph*(2″)-*Ig* (gentamicin); *aph*(3′)-*IIIa* and *aph*(3′)-*VIIa* (kanamycin); *ant*(9) (spectinomycin); *ant*(6)-*Ia* and *aadE*-Cc (streptomycin); and *sat*-4 (streptothricin) (Jacob et al., 1994; Zhao et al., 2015; Fabre et al., 2018). In general, the prevalence of all these aminoglycoside resistance genes was low in the *Campylobacter* genomes tested, ranging from 0.42% (1/237 genomes) to 5.06% (12/237 genomes), being the *aadE*-Cc and *aph*(3′)-*IIIa* genes those showing the highest prevalence; *ant*(6)-*Ia*, *ant*(9), *aph*(2″)-*If*, *aph*(2″)-*Ig*, *aph*(3′)-*VIIa* and *sat*-4 were found with a prevalence of 0.42, 0.42, 1.27, 1.69, 0.84, and 2.53%, respectively (Figure 1). It is worth mentioning that 5 additional genomes carried a truncated *ant*(6)-*Ia* gene and for this reason they were not considered in the final results; these truncated genes were located on plasmids forming a resistance cluster with the *sat*-4 and *aph*(3′)-*IIIa* genes (Figure 3B). In contrast, the full-length *ant*(6)-*Ia* gene was not found close to the *sat*-4 and *aph*(3′)-*IIIa* genes, instead, it was located close to the *tet*(O) gene (Figure 3B). Regarding the other aminoglycoside resistance genes, most of them were located on plasmids as single units, with exception of *aadE*-Cc (12/12 genomes) and *aph*(2″)-*If* (2/3 genomes) that were situated on chromosome (Table 1).

Additionally, our analysis revealed mutations in 3 housekeeping genes: *gyrA*, *rpsL*, and 23S rRNA (Figure 1), which confer resistance to fluoroquinolones (Wang et al., 1993; Charvalos et al., 1996), streptomycin (aminoglycosides) (Olkkola et al., 2010) and macrolides (Jensen and Aarestrup, 2001), respectively. Several point mutations in the *gyrA* gene have been associated with resistance to fluoroquinolones in *Campylobacter* spp., the predominant being T86I, caused by the C257T change in *gyrA;* other mutations include T86A, T86K, T86V, D90N, D90Y, A70T and the double mutation T86I/P104S and T86I/D90N (Wang et al., 1993; Bachoual et al., 2001; Hakanen et al., 2002; Piddock et al., 2003; McIver et al., 2004; Ruiz et al., 2005; Hanninen and Hannula, 2007; Tang et al., 2017). Mutations in *gyrA* were identified in 13.9% (33/237 genomes) of the *Campylobacter* genomes analyzed (Figure 1). These mutations are T86I (22/237 genomes), T86K (1/237 genomes), T86V (8/237 genomes), P104T (1/237 genomes) and the combination T86A/D90Y (1/237 genomes). On the other hand, in *C. coli* and *C. jejuni*, resistance to macrolides has been attributed to point mutations in domain V of the 23S rRNA gene, at positions 2074 and 2075 (A2074C, A2074G and A2075G), being the A2075G transition the most frequent in *Campylobacter* (Jensen and Aarestrup, 2001; Vacher et al., 2003; Ren et al., 2011). The bulk of *Campylobacter* macrolide-resistant isolates have mutations in the 3 copies of the 23S rRNA gene, but some present only 2 mutated copies (Gibreel et al., 2005). Our analysis revealed the presence of point mutations in the 23S rRNA gene in 2.53% (6/237 genomes) of the *Campylobacter* genomes tested (Figure 1), including A2074G (5/237 genomes) and A2074G/A2075G transitions (1/237 genomes). In 5 genomes, these point mutations were present in the 3 copies of the 23S rRNA gene, whereas in one genome, the A2074G mutation was found only in 2 copies of this gene. Finally, our analysis also revealed mutations in the *rpsL* gene, which codes for the S12 ribosomal protein, a component of the 30S ribosomal subunit. The K43R, K88E, K88Q and K88R mutations in RpsL have been associated with streptomycin resistance, being the K43R mutation the most commonly found in *Campylobacter* (Olkkola et al., 2010). We identified some of these mutations in RpsL, in 2.11% (5/237 genomes) of the *Campylobacter* genomes analyzed: K43R (1/237 genomes), K88R (3/237 genomes) and K43R/K88R (1/237 genomes) (Figure 1).

**FIGURE 4:**
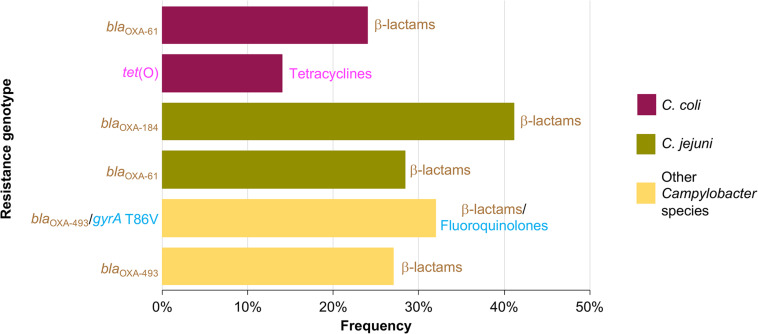
Frequency of the most prevalent antibiotic resistance genotypes on genomes from *C. coli*, *C. jejuni* and other *Campylobacter* species. Other *Campylobacter* species include: *C. avium*, *C. fetus*, *C. helveticus*, *C. hyointestinalis*, *C. iguaniorum*, *C. insulaenigrae*, *C. lanienae*, *C. lari*, *C. peloridis*, *C. sputorum*, *C. subantarcticus*, *C. ureolyticus*, and *C. volucris*. For each genotype, the antibiotic class to which they putatively confer resistance is indicated.

AR genotypes were identified according to the different combinations of the genetic determinants associated with AR found in the *Campylobacter* genomes analyzed. A total of 37 AR genotypes were recognized, being the AR genotypes conferring resistance to β-lactams the most predominant found. The different AR genotypes and their prevalence on genomes from *C. coli*, *C. jejuni*, and other *Campylobacter* species, are shown in Supplementary Figures S1–S3. *bla*_OXA–61_ and *tet*(O) were the most common AR genotypes present on genomes from *C. coli*, with a prevalence of 23.8% (5/21 genomes) and 14.3% (3/21 genomes), respectively; whereas *bla*_OXA–184_ (prevalence of 41.1%; 58/141 genomes) and *bla*_OXA–61_ (prevalence of 28.4%; 40/141 genomes) were the predominant AR genotypes on genomes from *C. jejuni*; and *bla*_OXA–493_/*gyrA* T86V and *bla*_OXA–493_ were the AR genotypes more common on genomes from other *Campylobacter* species, with a prevalence of 31.8% (7/22 genomes) and 27.3% (6/22 genomes), respectively (Figure 4). Location (chromosome or plasmid) of the AR determinants for some of the AR genotypes is shown in Figure 3B. All AR genotypes present in the *Campylobacter* plasmids are displayed in Figure 2.

### Genetic Determinants Associated With AR That Are *Campylobacter* Species-Specific

Interestingly, our analysis revealed that some genetic determinants for AR are present only in particular *Campylobacter* species. For instance, despite the great representativeness of genomes from *C. jejuni* (163 genomes), and with less abundance from *C. coli* (22 genomes), the *bla*_OXA–493_ gene was only found on genomes from 4 of the 6 species that integrate the *C. lari* group (Miller et al., 2014; Costa and Iraola, 2019): *C. insulaenigrae* (3/3 genomes), *C. lari* (7/8 genomes), *C. subantarcticus* (2/2 genomes) and *C. volucris* (1/1 genomes) (Supplementary File S1); the number of genomes carrying the *bla*_OXA–493_ gene with respect to the total of genomes tested for each species, is indicated between parenthesis. Hence, *bla*_OXA–493_ was the predominant gene for β-lactamases that was identified in the *C. lari* group (prevalence of 92.9%, 13/14 genomes); only one genome from this group presents the *bla*_OXA–184_ gene and none carries the *bla*_OXA–61_ or *bla*_OXA–576_ genes. In contrast, the *bla*_OXA–61_ gene was confined to *C. jejuni* (63/163 genomes) and *C. coli* (14/22 genomes) species, as well as the *bla*_OXA–184_ gene (66 genomes) was mostly present in *C. jejuni* (65/163 genomes); only one genome of *C. helveticus* also carries the *bla*_OXA–184_ gene (1/1 genomes) (Supplementary File S1). Furthermore, aminoglycoside resistance genes were only detected in *C. coli* (12/22 genomes) and *C. jejuni* (11/163 genomes) (Supplementary File S1).

On another hand, the T86V substitution in GyrA (with a prevalence of 24.2% with respect to the total of GyrA substitutions, 8/33 genomes) was detected only in species of the *C. lari* group: *C. insulaenigrae* (3/3 genomes), *C. lari* (1/8 genomes), *C. peloridis* (1/1 genomes), *C. subantarcticus* (2/2 genomes) and *C. volucris* (1/1 genomes) (Supplementary File S1). In contrast, the T86I mutation in GyrA (with a prevalence of 66.7% with respect to the total of GyrA substitutions, 22/33 genomes) was identified mostly in genomes of *C. jejuni* (18/22), but not in those from the *C. lari* group (Supplementary File S1).

### Prevalence of AR Genotypes in *Campylobacter* and Its Association With Collection Date, Host and Geographic Location

**FIGURE 5:**
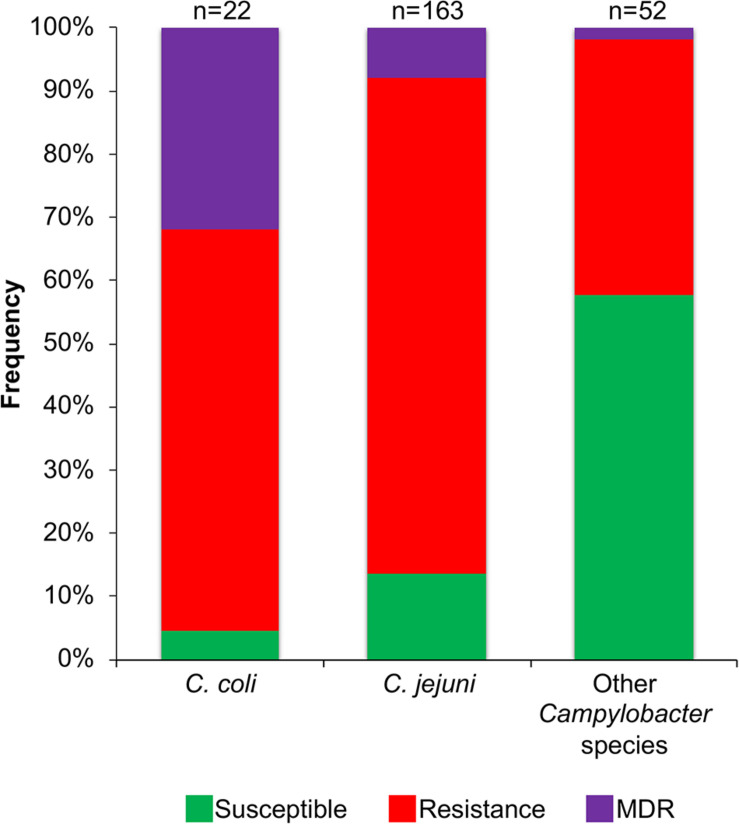
Frequency of susceptible, resistance or multidrug resistance (MDR) genotypes on the 237 *Campylobacter* genomes tested. Frequencies for genomes from each *C. coli*, *C. jejuni* and other *Campylobacter* species (*C. avium*, *C. concisus, C. cuniculorum*, *C. curvus*, *C. fetus*, *C. gracilis*, *C. helveticus*, *C. hepaticus*, *C. hominis*, *C. hyointestinalis*, *C. iguaniorum*, *C. insulaenigrae*, *C. lanienae*, *C. lari*, *C. peloridis*, *C. pinnipediorum*, *C. sputorum*, *C. subantarcticus*, *C. ureolyticus*, and *C. volucris*) are indicated. The genotypes were classified as follows: susceptible, lacks genetic determinants for antibiotic resistance; resistance, carries one or more genetic determinants related to resistance to antibiotics belonging to 1 or 2 different classes; MDR, contains one or more genetic determinants related to resistance to antibiotics belonging to 3 or more different classes. The number (n) of the respective genomes analyzed is shown above the columns.

Genetic factors associated with AR (genes or point mutations) were identified in 77.6% of the *Campylobacter* genomes analyzed (184/237 genomes; 1 to 8 factors per genome). No genetic determinants associated with AR were found on genomes from *C. concisus, C. cuniculorum*, *C. curvus*, *C. gracilis*, *C. hepaticus*, *C. hominis*, and *C. pinnipediorum*. Genomes carrying factors that putatively confer resistance to 1 or 2 classes of antibiotics were considered to represent a resistance genotype, those carrying factors related with resistance to 3 or more antibiotic classes were considered to be a multidrug resistance (MDR) genotype, and those lacking factors for AR were taken as susceptible genotypes. Most studies on AR in *Campylobacter* have been performed on the *C. coli* and *C. jejuni* species, whereas this phenomenon remains poorly explored for the other *Campylobacter* species. Thus, to gain more insight about the AR in *C. coli* and *C. jejuni*, as well as in the *Campylobacter* species less studied, we decided to compare the prevalence of resistance or MDR genotypes between genomes from *C. coli* (22 genomes), *C. jejuni* (163 genomes) and the other *Campylobacter* species analyzed (52 genomes). For the genomes from *C. coli*, the resistance genotype is the most represented (prevalence of 63.6%; 14/22 genomes), followed by the MDR genotype (prevalence of 31.8%; 7/22 genomes) and finally the susceptible genotype (prevalence of 4.6%; 1/22 genomes) (Figure 5). For genomes from *C. jejuni*, the resistance genotype is also the predominant (prevalence of 78.5%; 128/163 genomes), then the susceptible genotype (prevalence of 13.5%, 22/163 genomes) and at last the MDR genotype (prevalence of 8%; 13/163 genomes) (Figure 5). In contrast, for the genomes from the other *Campylobacter* species, the susceptible genotype is predominant (prevalence of 57.7%; 30/52 genomes), followed by the resistance genotype (prevalence of 40.4%; 21/52 genomes) and only one MDR genotype was found (prevalence of 1.9%; 1/52 genomes) (Figure 5).

**FIGURE 6:**
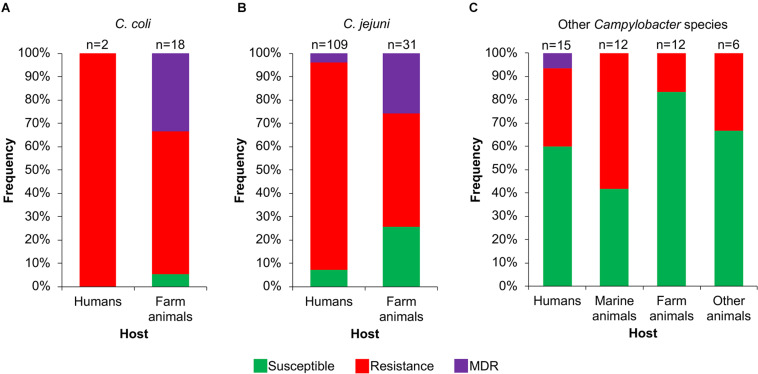
Frequency of susceptible, resistance or multidrug resistance (MDR) genotypes on *Campylobacter* genomes from isolates collected from different hosts. Genotypes from **(A)** *C. coli*, **(B)** *C. jejuni* and **(C)** other 20 *Campylobacter* species: *C. avium*, *C. concisus, C. cuniculorum*, *C. curvus*, *C. fetus*, *C. gracilis*, *C. helveticus*, *C. hepaticus*, *C. hominis*, *C. hyointestinalis*, *C. iguaniorum*, *C. insulaenigrae*, *C. lanienae*, *C. lari*, *C. peloridis*, *C. pinnipediorum*, *C. sputorum*, *C. subantarcticus*, *C. ureolyticus*, and *C. volucris*. Farm animals include chicken, bovine, pig, sheep, turkey, and rabbit; marine mammals comprise seal, sea lion, penguin, albatross, gull, and shellfish; other animals encompass iguana, lizard, snake, cat, and alpaca. Only those hosts with more available information are displayed. The number (n) of genomes corresponding to each host is shown above the columns.

**FIGURE 7:**
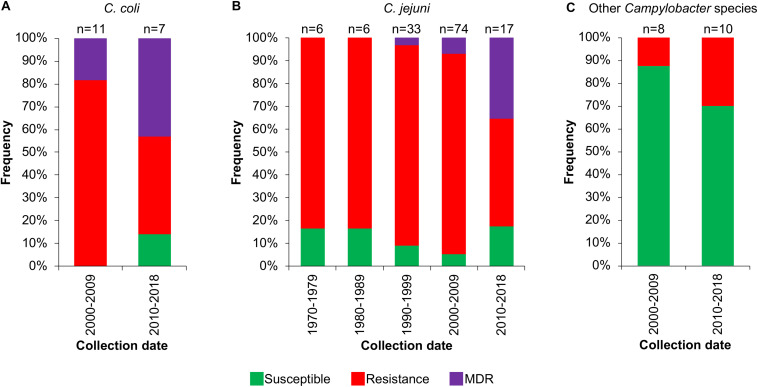
Frequency of susceptible, resistance or multidrug resistance (MDR) genotypes on *Campylobacter* genomes from isolates collected in different decades. Genotypes from **(A)** *C. coli*, **(B)** *C. jejuni* and **(C)** other 9 *Campylobacter* species: *C. avium*, *C. concisus, C. cuniculorum*, *C. fetus*, *C. hepaticus*, *C. iguaniorum*, *C. lari*, *C. pinnipediorum*, and *C. sputorum*. Only those decades with more available information are displayed. The number (n) of genomes corresponding to each decade is shown above the columns.

**FIGURE 8:**
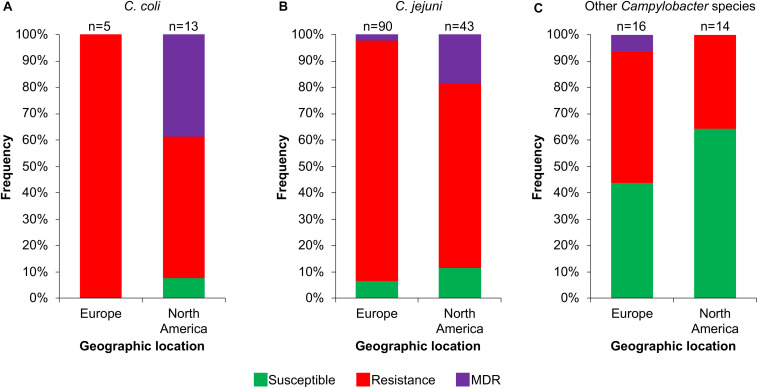
Frequency of susceptible, resistance or multidrug resistance (MDR) genotypes on *Campylobacter* genomes from isolates collected in different geographic locations. Genotypes from **(A)** *C. coli*, **(B)** *C. jejuni* and **(C)** other 18 *Campylobacter* species: *C. avium*, *C. concisus, C. cuniculorum*, *C. fetus*, *C. gracilis*, *C. helveticus*, *C. hepaticus*, *C. hyointestinalis*, *C. iguaniorum*, *C. insulaenigrae*, *C. lanienae*, *C. lari*, *C. peloridis*, *C. pinnipediorum*, *C. sputorum*, *C. subantarcticus*, *C. ureolyticus*, and *C. volucris*. Europe includes Belgium, Denmark, Finland, France, Germany, Italy, Netherlands, Sweden, Switzerland, and United Kingdom; North America comprises Canada and United States. Only those geographic locations with more available information are displayed. The number (n) of genomes corresponding to each geographic location is shown above the columns.

Then, we analyzed the prevalence of the *Campylobacter* AR genotypes according to the host, collection date and geographical location from where the respective bacteria were isolated (Supplementary File S1). The *C. coli* isolates from farm animals carry resistance or MDR genotypes, with prevalence of 61.1% (11/18 genomes) and 33.3% (6/18 genomes), respectively, whereas the only 2 *C. coli* isolates from humans present the resistance genotype (Figure 6A). For *C. jejuni*, the MDR genotype has a higher prevalence in isolates from farm animals (25.8%; 8/31 genomes) compared with those from humans (3.7%; 4/109 genomes); the opposite was observed for the resistance genotype (Figure 6B). For the other *Campylobacter* species, the resistance genotype is predominant in bacteria isolated from marine animals (58.3%; 7/12 genomes), whereas the only MDR genotype found in this group is present in the *C. lanienae* NCTC 13004 strain isolated from a human (6.7%; 1/15 genomes) (Figure 6C). Notably, our analyses show a considerable increase in the presence of MDR genotypes from *C. coli* (Figure 7A) and *C. jejuni* (Figure 7B), as well as in the presence of resistance genotypes from the other *Campylobacter* species group (Figure 7C), in bacteria collected in the last decade (2010–2018), compared with previous decades. Additionally, our results indicate a very high prevalence of AR genotypes in *C. coli* and *C. jejuni* isolates from both Europe and North America (88–100%); being the MDR genotype from both *C. coli* and *C. jejuni* more predominant in North America (Figures 8A,B). Likewise, our analyses indicate a higher prevalence of AR genotypes from the other *Campylobacter* species group in Europe (56.3%) with respect to North America (35.7%) (Figure 8C).

## Discussion

Previous studies have analyzed the prevalence of genetic determinants for AR in genomes from *C. coli* and *C. jejuni* strains isolated in specific geographic regions (Weis et al., 2016; Zhao et al., 2016; Cantero et al., 2018; de Vries et al., 2018; Fabre et al., 2018; Whitehouse et al., 2018). In the present study, we identified genetic determinants associated with AR in 237 genomes from 22 different *Campylobacter* species with global distribution.

We found 15 distinct AR genes and point mutations related to AR in 3 housekeeping genes in the *Campylobacter* genomes tested (Figure 1). Notably, 3 of the AR genes had not been previously reported in *Campylobacter*: *lnu*(AN2), putatively associated with lincosamides resistance, and 2 putative β-lactams resistance genes, *bla_OXA–493_* and *bla_OXA–576_*. Two β-lactamases coding genes, *bla*_OXA–61_ and *bla*_OXA–184_, were the most abundant AR genes in the analyzed genomes from *C. coli* and *C. jejuni*, which is consistent with the results from previous studies (Griggs et al., 2009; Weis et al., 2016; Zhao et al., 2016; de Vries et al., 2018; Fabre et al., 2018; Whitehouse et al., 2018). In contrast, in the analyzed genomes from species other than *C. coli* and *C. jejuni*, the *bla*_OXA–493_ β-lactamase coding gene was the predominant (Supplementary File S1). On another hand, even though our study indicates a low occurrence of determinants associated with aminoglycoside resistance in the *Campylobacter* genomes tested, these determinants displayed a great diversity, since 9 different aminoglycoside resistance genes, as well as point mutations in *rpsL*, were found (Figure 1). These findings are in agreement with those reported previously (Qin et al., 2012; Zhao et al., 2015, 2016; Cantero et al., 2018; Fabre et al., 2018; Hormeno et al., 2018; Whitehouse et al., 2018).

A high global incidence of *Campylobacter* isolates resistant to ciprofloxacin (fluoroquinolones) and erythromycin (macrolides) has been reported before (Luangtongkum et al., 2009; Signorini et al., 2018; Sproston et al., 2018). However, our analysis showed a low occurrence of mutations in the *gyrA* and 23S rRNA genes (Figure 1), which are associated with resistance to fluoroquinolones and macrolides, respectively. Nevertheless, multidrug efflux pumps can also mediate resistance to antibiotics of different classes, such as aminoglycosides, β-lactams, chloramphenicol, fluoroquinolones, macrolides and tetracyclines, among others (Blair et al., 2014). The efflux pumps are ancient elements that play important physiological roles in bacteria; but notably, they can also export antibiotics out of the cell, thus reducing their intracellular concentration and, as consequence, confer AR (Blair et al., 2014; Blanco et al., 2016). CmeABC is the only multidrug efflux pump characterized in *Campylobacter*; it was shown to be involved in resistance to structurally different antibiotics including β-lactams, fluoroquinolones, macrolides, and tetracyclines (Lin et al., 2002; Luangtongkum et al., 2009; Guo et al., 2010; Shen et al., 2018). In addition to specific genes and point mutations for AR, we also detected genes that code for multiple efflux pumps in *Campylobacter* (Supplementary File S2). Genes for the CmeABC system, MacB family, macrolide ABC transporter and TolC family efflux pumps, were found in the bulk of analyzed genomes. Besides, genes for other efflux pumps were found only in some genomes, such as those for the AcrB/AcrD/AcrF family, hydrophobe/amphiphile efflux-1 family and multidrug efflux SMR transport efflux pumps. Important to note, given that the efflux pumps can confer resistance to multiple antibiotics, to facilitate interpretation, they were not considered in our analysis as determinants for AR.

A major finding of our work was the *Campylobacter* species-specific distribution of some genetic determinants for AR. *Campylobacter* species cluster in 5 discrete phylogenetic clades (Costa and Iraola, 2019). One is the *C. lari* group, which is composed of six species: *C. insulaenigrae*, *C. lari*, *C. ornithocola*, *C. peloridis*, *C. subantarcticus*, and *C. volucris* (Miller et al., 2014; Costa and Iraola, 2019). These species are highly related at the genome level and have been isolated from similar hosts and environments (Miller et al., 2014). We found the *bla*_OXA–493_ gene and the T86V substitution in GyrA, exclusively in species from the *C. lari* group (Supplementary File S1). The T86V mutation in GyrA had also been previously reported in the *C. lari* species (Piddock et al., 2003; Weis et al., 2016). In contrast, aminoglycoside resistance genes were only detected in the classical *C. coli* and *C. jejuni* species (Supplementary File S1). Previous studies also have indicated the presence of aminoglycoside resistance genes in *C. jejuni* and *C. coli* (Qin et al., 2012; Zhao et al., 2015, 2016; Cantero et al., 2018; Fabre et al., 2018; Whitehouse et al., 2018); however, the presence of these genes in species other than *C. jejuni* and *C. coli* had not been analyzed. Thus, our data show that the genetic variability between the different clades of *Campylobacter* also involves the determinants for AR. Due to the limited number of genomes from non-*C. coli*/*C. jejuni* species (52 genomes), we cannot discard the possibility that aminoglycoside resistance genes are present in these species, but in very low prevalence.

Consistent with previous reports (NARMS, 2012; de Vries et al., 2018; Whitehouse et al., 2018), we found that the *C. coli* genomes harbor the highest number of AR determinants, compared with the other *Campylobacter* species analyzed (Supplementary File S1). Furthermore, in agreement with previous studies (Thakur et al., 2010; Wieczorek et al., 2015), our results show a higher prevalence of MDR genotypes in *C. coli* (31.8%; 7/22 genomes) with respect to *C. jejuni* (8%; 13/163 genomes). Notably, 86.4% (19/22 genomes) of the *C. coli* genomes possess 1–4 plasmids; in contrast, only 14.1% (23/163 genomes) of the *C. jejuni* genomes carry 1 or 2 plasmids, and 25% (13/52 genomes) of the genomes from the remaining 20 *Campylobacter* species tested contain 1–5 plasmids (Supplementary File S1). Interestingly, most genes associated with AR in *C. coli* are located on plasmids, whereas in the other *Campylobacter* species are placed on chromosome (Supplementary File S1), which could explain, at least in part, the higher number of AR determinants and MDR genotypes in *C. coli* compared with the other *Campylobacter* species. Remarkably, in non-*C. coli*/*C. jejuni* species, AR genes located on plasmids were not identified (Figure 2 and Supplementary File S1), *Campylobacter* spp. have mechanisms for conjugation and natural transformation, and transferrable AR has been documented in this genus (Taylor and Courvalin, 1988). Moreover, it has been proposed that *Campylobacter* gained some AR genes from Gram-positive bacteria (Taylor and Courvalin, 1988; Zilhao et al., 1988; Alfredson and Korolik, 2007). Thus, the AR genes located on plasmids, or on other mobile elements, imply a threat for the appearance of new *Campylobacter* strains resistant to antibiotics.

We found a reduced number of genetic determinants for AR in *Campylobacter*, in comparison with the huge number of genetic determinants for AR present in other bacteria such as *Enterococcus* spp. (Torres et al., 2018), *Escherichia coli* (Poirel et al., 2018) or *Salmonella enterica* (McDermott et al., 2018), which share hosts and niches with *Campylobacter* spp. The reason why *Campylobacter* spp. maintain in general a low number of genetic determinants for AR remains to be determined.

Our results, together with previous studies, reveal a high prevalence of *Campylobacter* AR genotypes worldwide, not only from the classical *C. jeuni* and *C. coli* species, but also, although still with less extent, from emerging *Campylobacte*r species; these AR genotypes can be present in bacteria residing in different hosts such as humans and different animals (Figures 5,6,8 and Supplementary File S1). Even more worryingly, our data support that the prevalence of these bacteria carrying AR genotypes has been increasing with time (Figure 7).

Although the phenotype conferred by all the resistance genetic determinants identified in this study needs to be tested, a very high correlation between the presence of genetic determinants for AR and the respective phenotype has been reported in *Campylobacter* spp., which reaches up to 100% of correspondence for some specific antibiotics (Nirdnoy et al., 2005; Chen et al., 2013; Zhao et al., 2015, 2016; Fabre et al., 2018; Whitehouse et al., 2018). Hence, it has been suggested that analysis of genomic data, for the identification of genetic determinants associated with AR, has the potential to reliably predict resistance phenotypes (Zhao et al., 2016; Whitehouse et al., 2018; Feldgarden et al., 2019).

Thus, our study, together with other reports, provide genetic determinants that can be used to predict AR in *Campylobacter* spp., which could greatly help to select the best antibiotic therapy against infections caused by these bacteria. Additionally, our study further expands the knowledge on the genetic elements that shape the resistome of the genus *Campylobacter* and on the scattering of these AR genetic determinants between the *Campylobacter* species.

## Data Availability Statement

All datasets generated for this study are included in the article/Supplementary Material.

## Author Contributions

DP-M and VB contributed to the conception and design of the study, as well as the discussion of the results. DR-M, IM-F, RS, and LL carried out the bioinformatics work. DP-M analyzed the bioinformatics results, created the figures, and wrote the manuscript. VB edited the manuscript. All authors contributed to the article and approved the submitted version.

## Conflict of Interest

The authors declare that the research was conducted in the absence of any commercial or financial relationships that could be construed as a potential conflict of interest.

## Footnotes

1 www.bacterio.net/campylobacter.html

2 https://www.who.int/news-room/fact-sheets/detail/campylobacter

3 https://www.ncbi.nlm.nih.gov/refseq/

4 https://card.mcmaster.ca/

5 https://www.ncbi.nlm.nih.gov/pathogens/antimicrobial-resistance/

6 https://blast.ncbi.nlm.nih.gov/Blast.cgi

7 https://cge.cbs.dtu.dk/services/ResFinder/

8 https://www.megasoftware.net

## Supplementary Material

FIGURE S1 Frequency of resistance or multidrug resistance (MDR) genotypes on *C. coli* genomes. Thirteen antibiotic resistance genotypes were detected in 21 genomes from *C. coli* isolates. For each genotype, the antibiotic class to which they putatively confer resistance is indicated.

FIGURE S2 Frequency of resistance or multidrug resistance (MDR) genotypes on *C. jejuni* genomes. Twenty antibiotic resistance genotypes were detected in 141 genomes from *C. jejuni* isolates. For each genotype, the antibiotic class to which they putatively confer resistance is indicated.

FIGURE S3 Frequency of resistance or multidrug resistance (MDR) genotypes on genomes from other *Campylobacter* species. Eleven antibiotic resistance genotypes were detected in 22 genomes from other *Campylobacter* species isolates: *C. avium*, *C. fetus*, *C. helveticus*, *C. hyointestinalis*, *C. iguaniorum*, *C. insulaenigrae*, *C. lanienae*, *C. lari*, *C. peloridis*, *C. sputorum*, *C. subantarcticus*, *C. ureolyticus*, and *C. volucris*. For each genotype, the antibiotic class to which they putatively confer resistance is indicated.
