# Supplementary figures and images for: Genomic Analysis Reveals the Genetic Determinants Associated With Antibiotic Resistance in the Zoonotic Pathogen Campylobacter spp. Distributed Globally
Source: Front Microbiol. 2020 Sep 11;11:513070. doi: 10.3389/fmicb.2020.513070 (PMC7518152; doi:10.3389/fmicb.2020.513070)

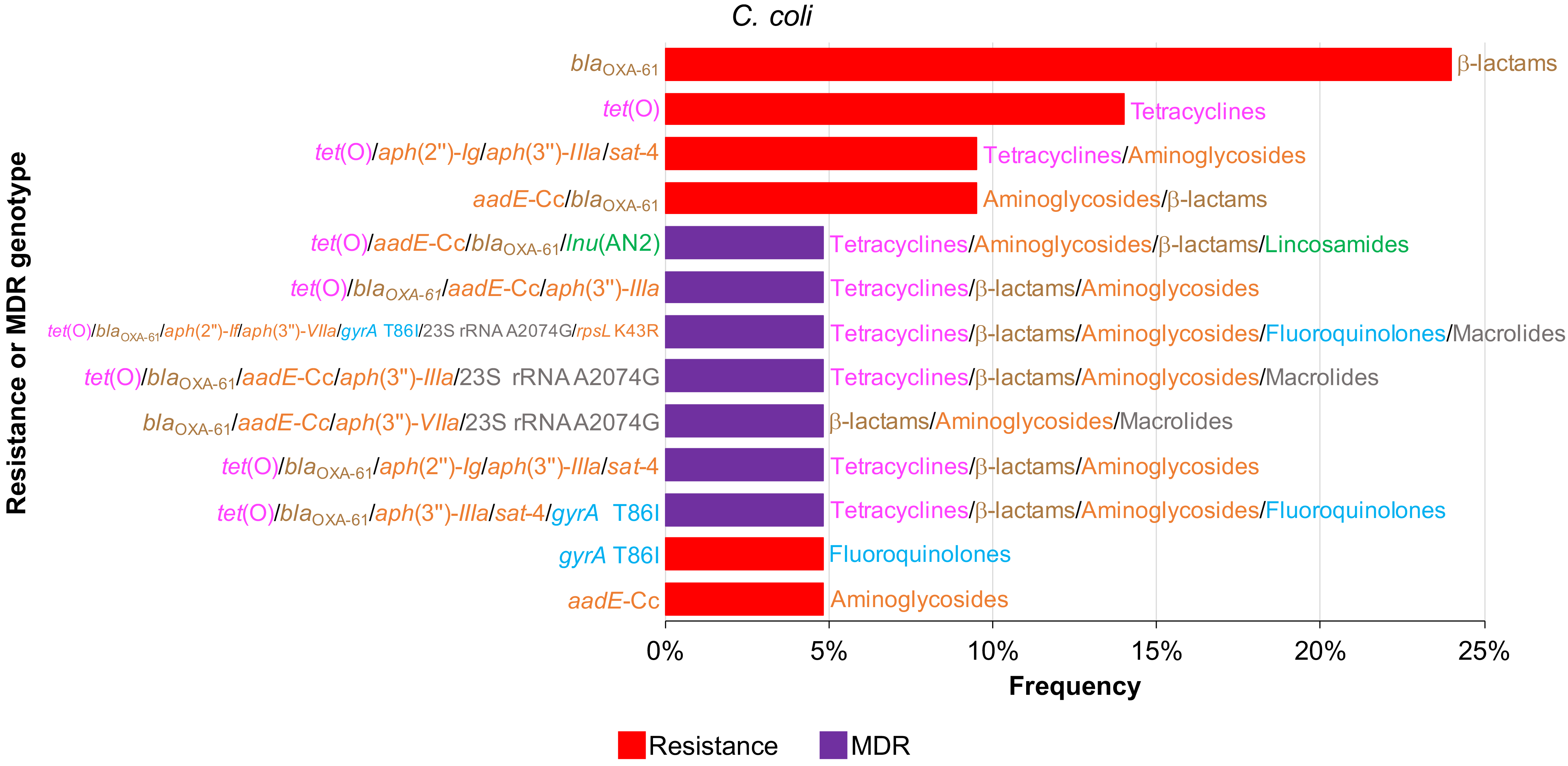

Supplement: FIGURE S1 — Frequency of resistance or multidrug resistance (MDR) genotypes on C. coli genomes. Thirteen antibiotic resistance genotypes were detected in 21 genomes from C. coli isolates. For each genotype, the antibiotic class to which they putatively confer resistance is indicated. [file Image_1.TIF]

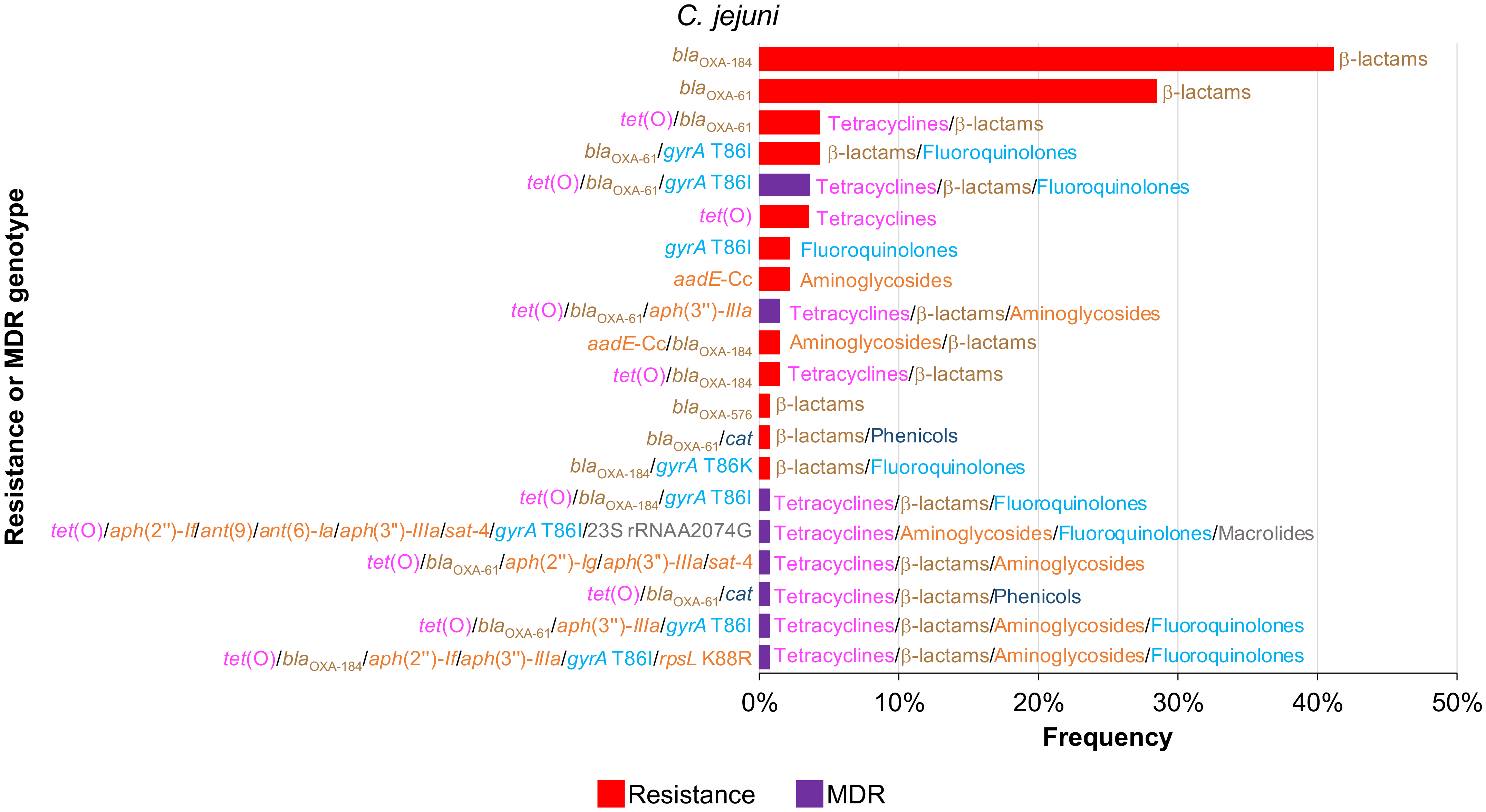

Supplement: FIGURE S2 — Frequency of resistance or multidrug resistance (MDR) genotypes on C. jejuni genomes. Twenty antibiotic resistance genotypes were detected in 141 genomes from C. jejuni isolates. For each genotype, the antibiotic class to which they putatively confer resistance is indicated. [file Image_2.TIF]

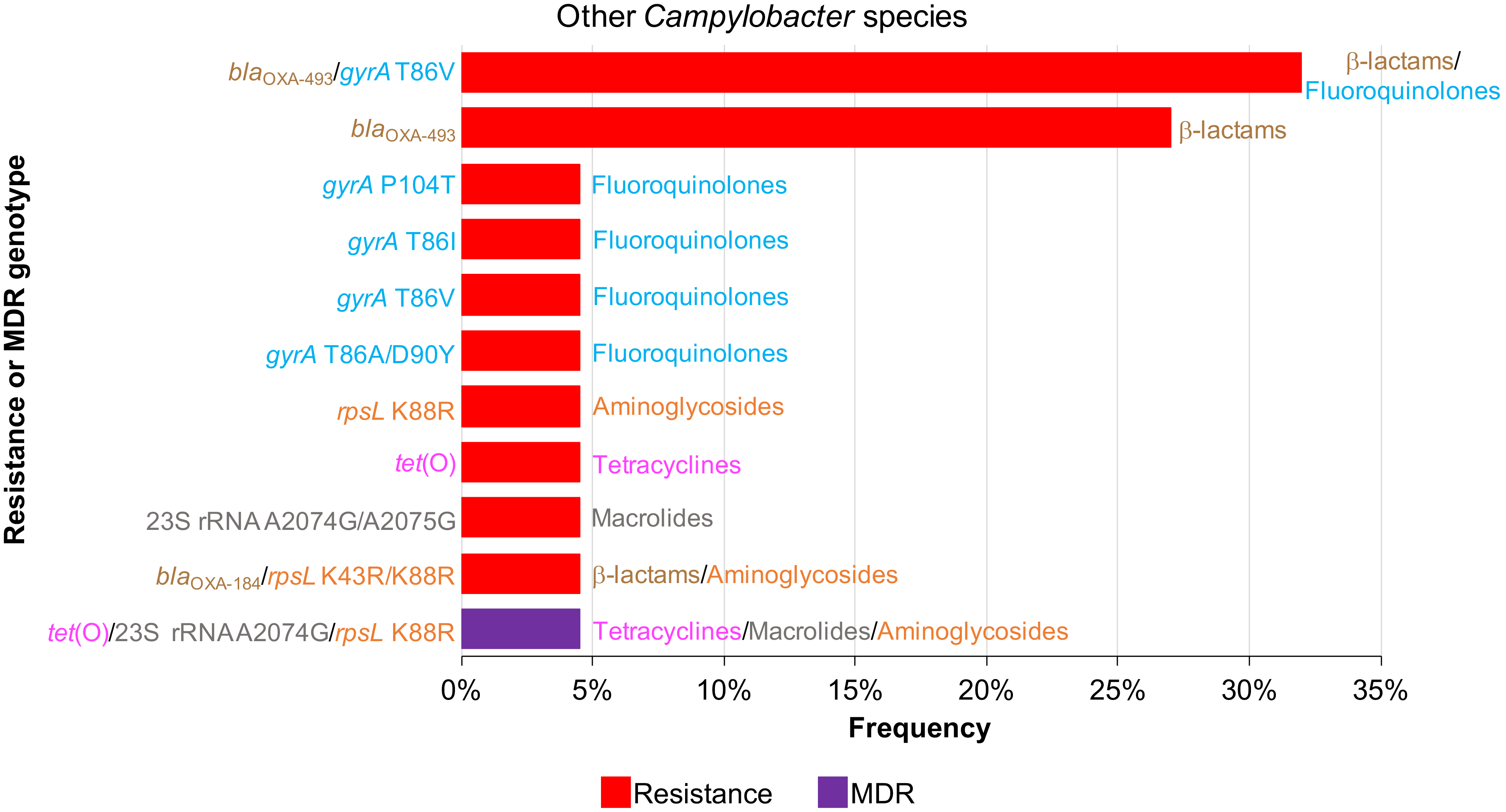

Supplement: FIGURE S3 — Frequency of resistance or multidrug resistance (MDR) genotypes on genomes from other Campylobacter species. Eleven antibiotic resistance genotypes were detected in 22 genomes from other Campylobacter species isolates: C. avium, C. fetus, C. helveticus, C. hyointestinalis, C. iguaniorum, C. insulaenigrae, C. lanienae, C. lari, C. peloridis, C. sputorum, C. subantarcticus, C. ureolyticus, and C. volucris. For each genotype, the antibiotic class to which they putatively confer resistance is indicated. [file Image_3.TIF]
